# Identifying Predictive Biomarkers of Subclinical Mastitis in Dairy Cows through Urinary Metabotyping

**DOI:** 10.3390/metabo14040205

**Published:** 2024-04-04

**Authors:** Grzegorz Zwierzchowski, Klevis Haxhiaj, Roman Wójcik, David S. Wishart, Burim N. Ametaj

**Affiliations:** 1 Department of Agricultural, Food and Nutritional Science, University of Alberta, Edmonton, AB T6G 2P5, Canada; grzegorz.zwierzchowski@uwm.edu.pl (G.Z.); haxhiaj@ualberta.ca (K.H.); 2 Faculty of Biology and Biotechnology, University of Warmia and Mazury, 1a Oczapowskiego Str., 10-719 Olsztyn, Poland; 3 Faculty of Veterinary Medicine, University of Warmia and Mazury, 1a Oczapowskiego Str., 10-719 Olsztyn, Poland; brandy@uwm.edu.pl; 4 Department of Biological and Computer Sciences, University of Alberta, Edmonton, AB T6G 2P5, Canada; dwishart@ualberta.ca

**Keywords:** mastitis biomarkers, urine metabolomics, dairy cow, metabotyping, DI/LC-MS/MS

## Abstract

Mastitis is a significant infectious disease in dairy cows, resulting in milk yield loss and culling. Early detection of mastitis-prone cows is crucial for implementing effective preventive measures before disease onset. Current diagnosis of subclinical mastitis (SCM) relies on somatic cell count assessment post-calving, lacking predictive capabilities. This study aimed to identify metabolic changes in pre-SCM cows through targeted metabolomic analysis of urine samples collected 8 wks and 4 wks before calving, using mass spectrometry. A nested case-control design was employed, involving a total of 145 multiparous dairy cows, with disease occurrence monitored pre- and postpartum. Among them, 15 disease-free cows served as healthy controls (CON), while 10 cows exclusively had SCM, excluding those with additional diseases. Urinary metabolite profiling revealed multiple alterations in acylcarnitines, amino acids, and organic acids in pre-SCM cows. Metabotyping identified 27 metabolites that distinguished pre-SCM cows from healthy CON cows at both 8 and 4 wks before parturition. However, only four metabolites per week showed significant alterations (*p* < 0.005). Notably, a panel of four serum metabolites (asymmetric dimethylarginine, proline, leucine, and homovanillate) at 8 wks prepartum, and another panel (asymmetric dimethylarginine, methylmalonate, citrate, and spermidine) at 4 wks prepartum, demonstrated predictive ability as urinary biomarkers for SCM risk (AUC = 0.88; *p* = 0.02 and AUC = 0.88; *p* = 0.03, respectively). In conclusion, our findings indicate that metabolite testing can identify cows at risk of SCM as early as 8 and 4 wks before parturition. Validation of the two identified metabolite panels is warranted to implement these predictive biomarkers, facilitate early intervention strategies, and improve dairy cow management to mitigate the impact of SCM. Further research is needed to confirm the efficacy and applicability of these biomarkers in practical farm settings.

## 1. Introduction

Bovine mastitis, a major concern for the dairy industry, is characterized by udder inflammation [1]. Various factors, including bacterial infection, poor farm hygiene, dry cow therapy, or automated milking machines, contribute to its pathogenesis [1]. The estimated cost of mastitis is $660 per case in Canada and $2.5 billion in North America annually [2]. Mastitis can manifest as clinical or subclinical forms. Subclinical mastitis (SCM) is an asymptomatic udder inflammation characterized by the influx of cellular elements, primarily polymorphonuclear neutrophils, into the mammary gland [1]. On the other hand, clinical mastitis (CM) presents external changes to the udder (e.g., swelling, heat) and milk appearance (e.g., discoloration, thickness), along with systemic signs in the cow (e.g., fever, reduced feed intake) [1]. Udder infections lead to reduced milk production in current and subsequent lactations, as well as compromised reproductive performance and conception rates, resulting in early culling of dairy cows. In fact, mastitis is the second most common reason for culling dairy cows in Canada [1].

The traditional diagnostic method for subclinical mastitis is the somatic cell count (SCC) test, which assesses immune cell count, primarily neutrophils, in milk [3]. This test is conducted shortly after calving and throughout the milking period. However, a recent study revealed a high incidence of new intramammary infections (IMI) during the dry period [3]. Subclinical IMI during dry-off may persist or develop into acute or subclinical mastitis following calving. Our findings indicate that cows infected after parturition exhibit a low-grade systemic inflammatory state during the dry-off period [4]. Additionally, our previous studies demonstrated multiple changes in serum and urinary metabolic signatures distinguishing SCM cows from healthy counterparts [5,6].

Metabolomics and microbiomics approaches have gained prominence in studies of periparturient diseases in dairy cows over the past decade. Most studies have focused on postpartum biofluids, such as milk or blood, for diagnostic purposes [7]. Limited data exists on urine metabolite fingerprinting in dairy cows to identify mastitis biomarkers or predict susceptibility. In a previous study, we examined urine samples from prepartum cows that later developed SCM postpartum, revealing urinary alterations in acylcarnitines (ACs), phosphatidylcholines (PCs), biogenic amines (BAs), and amino acids (AAs) from −8 wks to +8 wks around calving [6].

Recent reports indicate that UK farmers prefer test results within 24 h of sampling [8], while the demand for lab-on-chip or pen-side tests continues to grow. Hence, our laboratory focuses on identifying metabolites that differentiate healthy cows from those susceptible to SCM, aiming to develop pen-side tests utilizing these biomarkers.

Our hypothesis proposes that cows susceptible to SCM would present detectable urinary metabolite alterations starting at 8 wks and 4 wks prepartum, enabling the identification of susceptible cows during the dry-off period. This study, part of a larger project [9], employs a quantitative, targeted metabolomics approach to analyze urine samples from dairy cows. The objective is to identify urinary metabolites and assess whether those metabolites can be used for screening purposes as a pen-side test to predict the risk of SCM during the dry-off period.

## 2. Materials and Methods

### 2.1. Animals, Diets, and Urine Samples

In this nested case-control study, urine samples were collected from 145 multiparous cows on a commercial dairy farm in Alberta, Canada. The University of Alberta Animal Care and Use Committee for Livestock approved all experimental procedures, which were conducted in accordance with the Canadian Council’s guidelines on Animal Care (protocol number AUP00003216).

Cows were chosen based on their anticipated calving date and were sampled at the onset and midpoint of the dry-off period, corresponding to 8 wks (55–58 days) and 4 wks (27–30 days) before parturition, respectively. Throughout this paper, these samples will be referred to as −8 wks and −4 wks. Urine samples were collected with precision using the free catch method. The collection was conducted between 7:00 and 8:00 a.m., utilizing sterile 50 mL specimen tubes sourced from Fisher Scientific, Toronto, ON, Canada. To facilitate the collection, a gentle stimulation technique was employed. This involved cautiously rubbing the perineal area of the cows to induce urination, ensuring minimal stress and discomfort to the animals. The Metabolomics Innovation Centre (University of Alberta, Edmonton, AB, Canada) conducted all urinary metabolite analyses. All samples were stored at −80 °C until analysis.

The prepartum cows exhibited various conditions, such as mastitis, metritis, retained placenta, laminitis, displaced abomasum, milk fever, and postpartum ketosis. Health records for periparturient diseases were collected from the farm’s database. A herd veterinarian diagnosed all diseases during weekly visits to the herd. Positive cases for subclinical mastitis (SCM) were defined as cows with two or more consecutive wks of postpartum milk somatic cell counts (SCCs) equal to or exceeding 200,000 cells/mL. Cows without any health issues throughout the dry period and up to 4 wks postpartum, with SCCs below 200,000 cells/mL, were considered healthy controls (CON). Out of the initial group of 145 dairy cows, 44 were identified with high Somatic Cell Counts (SCC) exceeding 200,000 cells/mL of milk. Among these, 34 cows were diagnosed with Subclinical Mastitis (SCM) and also presented concurrent diseases. For this analysis, cows exhibiting other periparturient conditions such as metritis, retained placenta, laminitis, displaced abomasum, milk fever, and postpartum ketosis, which did not meet the specific criteria for comparison between SCM and control groups (CON), were excluded. Consequently, 15 cows were classified as healthy (CON), and only 10 met the criteria of having SCC indicative of subclinical mastitis without the presence of other diseases. During the two sampling wks, the body condition score (BCS) was determined for both groups (8 wks and 4 wks prepartum). Table 1 and Table 2 present the dry matter content of feed ingredients before and after calving.

### 2.2. Mass Spectrometry (MS)-Based Compound Identification and Quantification

#### 2.2.1. Sample Preparation

A targeted MS-based metabolomics method (TMIC Prime assay, PMID: 32634308) was utilized to analyze all urine samples. This method combines liquid chromatography (LC) and flow injection analysis (FIA) with an AB SCIEX QTRAP^®^ 4000 mass spectrometer (Framingham, MA, USA), enabling the identification and quantification of up to 142 different metabolites. To facilitate the identification and quantification of biogenic amines and organic acids, the samples were derivatized using 13C-labeled phenylisothiocyanate (PITC) and 3-nitrophenylhydrazine (3-NPH), respectively. Prior to MS analysis, the urine samples were thawed on ice and vortexed. For the analysis of biogenic amines, amino acids, lipids, acylcarnitines, and hexose (according to the manufacturer 95% glucose), 10 μL of flow injection analysis (FIA) running buffer and LC internal standards (ISTD) were loaded into a 96-well filter plate. The first 14 wells of the plate were utilized for quality control and standardization, including a blank, three zero samples, seven standards, and three quality control (QC) samples. The remaining 82 wells were allocated for the thawed urine samples, with 10 μL of urine added to each well for both the samples and standards. Subsequently, the plate was incubated and dried for 30 min using a nitrogen flow Zanntek Analytical Evaporator (Glas-Col, LLC, Terre Haute, IN, USA). After drying, each well received 50 μL of 5% phenylisothiocyanate (PITC) solution and was incubated at room temperature for 20 min. Following another 90-min drying period under nitrogen flow, the metabolites were extracted using 300 μL of methanol containing 5 mM ammonium acetate.

The plate was shaken at 330 rpm for 30 min, then centrifuged for 5 min at 500 rpm in a lower 96 deep-well plate using a Sorvall Evolution RC Superspeed Centrifuge (Fisher Scientific, Toronto, ON, Canada). The extract was diluted 1:1 with water for the analysis of amino acids and biogenic amines, and 10 μL of the diluted extract was injected into the LC column. Additionally, 150 μL of the extract was diluted with 400 μL of FIA running buffer for the analysis of acylcarnitines, lipids, and hexose compounds, and 20 μL of the diluted extract was injected into the LC column.

To each well, three solutions were added, as follows: (1) 25 μL of a 250 mM 3-nitrophenylhydrazine (3-NPH) solution in 50% aqueous methanol, (2) 25 μL of a 150 mM 1-ethyl-3-(3-dimethyl aminopropyl) carbodiimide solution in methanol, and (3) 25 μL of a 7.5% solution in 75% aqueous methanol. Subsequently, the plate was placed on a shaker and shaken at 450 rpm for 2 h at room temperature to allow for complete derivatization. After the reaction, each sample well received 350 μL of HPLC water and 50 μL of butylated hydroxytoluene (BHT) at a concentration of 2 mg/mL to dilute and stabilize the solution for LC-MS analysis.

#### 2.2.2. Tandem Mass Spectrometry (MS/MS)

Urine metabolites underwent separation and analysis using an Agilent 1100 series liquid chromatographic system (LC) (Agilent, Palo Alto, CA, USA) equipped with an Agilent reversed-phase Zorbax Eclipse XDB C18 column (3.0 mm × 100 mm, 3.5 M particle size, 80 Å pore size), along with a Phenomenex (Torrance, CA, USA) SecurityGuard C18 pre-column (4.0 mm × 3.0 mm), coupled with an AB SCIEX QTRAP^®^ 4000 mass spectrometer (Sciex Canada, Concord, ON, Canada).

LC-MS grade formic acid and high-performance liquid chromatography (HPLC) grade water were supplied by Fisher Scientific (Ottawa, ON, Canada), while ammonium acetate, phenylisothiocyanate (PITC), and HPLC grade acetonitrile (ACN) were provided by Sigma-Aldrich (St. Louis, MO, USA). The LC-MS assay workflow was managed using the Analyst^®^ 1.6.2 software (Sciex Canada, ON, Canada).

For analyzing amino acids and biogenic amines, the following HPLC parameters were utilized: mobile phase A containing 0.2% (*v*/*v*) formic acid in HPLC grade water and mobile phase B containing 0.2% (*v*/*v*) formic acid in ACN. The gradient profile for the HPLC solvent run was as follows: t = 0 min, 0% B; t = 0.5 min, 0% B; t = 5.5 min, 95% B; t = 6.5 min, 95% B; t = 7.0 min, 0% B; and t = 9.5 min, 0% B. The column oven temperature was set to 50 °C, with a sample injection volume of 10 μL and a flow rate of 500 μL/min. The mass spectrometer operated in positive electrospray ionization (ESI) mode with a scheduled multiple reaction monitoring (MRM) scan.

For FIA-MS/MS analysis, the HPLC autosampler was directly connected to the MS ion source using red PEEK tubing. The FIA running buffer served as the mobile phase, and the flow rate was programmed as follows: t = 0 min, 30 μL/min; t = 1.6 min, 30 μL/min; t = 2.4 min, 200 μL/min; t = 2.8 min, 200 μL/min; t = 3.0 min, 30 μL/min. The sample injection volume was 20 μL. The mass spectrometer operated in positive ESI mode with MRM scanning for analyzing lipids and acylcarnitines, while the negative ESI mode was employed for detecting glucose/hexose.

### 2.3. Statistical Analysis

Univariate analyses were conducted using the Wilcoxon rank sum test via the emmeans package in R (version 4.3.1), adhering to a significance threshold of *p* < 0.05. For metabolomic data, including multivariate and biomarker analyses, we employed MetaboAnalyst, following the established protocols [10,11]. The dataset was normalized using creatinine as a reference feature and underwent transformation and scaling to achieve a Gaussian distribution.

We conducted multivariate statistical analyses between the two cow groups, SCM and CON, using both unsupervised and supervised methods, including principal component analysis (PCA) and partial least squares discriminant analysis (PLSDA). In these analyses, the most influential compounds are typically ranked using variable importance in projection (VIP) plots, with metabolites demonstrating *p* < 0.05 and VIP > 1 being considered key discriminators between the groups.

Additionally, model validation was performed using a cross-validation test, and a 2000 permutation test was implemented to confirm the model’s reliability [12]. Biomarker profiles and metabolite set enrichment analyses (MSEA) were also conducted using MetaboAnalyst. The quality of the biomarker sets was assessed using receiver operator characteristic analysis (ROC, v4.0), generated through Monte Carlo cross-validation (MCCV). A permutation test with 1000 repeats was employed to validate these ROC curves.

For biomarker analysis, we selected top metabolites of high importance and constructed area under the curve (AUC) analyses for both time points. Linear and logistic regression analyses were applied to several significant metabolites. Finally, perturbed metabolic pathways identified from MSEA were deemed statistically significant if they met a Holm-corrected *p* value of <0.05.

## 3. Results

A total of 82 urinary metabolites were consistently identified and measured in all cows in this study. Our findings demonstrated significant differences in urinary metabolites between pre-SCM cows and healthy cows at 8 wks and 4 wks before parturition. At each sampling time (8 wks and 4 wks prepartum), 27 metabolites distinguished the pre-SCM only and CON groups, as shown in Table 3 and Table 4. Only four metabolites from each week exhibited FDR-adjusted *p* values below q < 0.005. Pre-SCM cows had a body condition score (BCS) of 3.70 at 8 wks and 3.92 at 4 wks, while control cows had a BCS of 3.78 at 8 wks and 3.95 at 4 wks.

**Figure 1:**
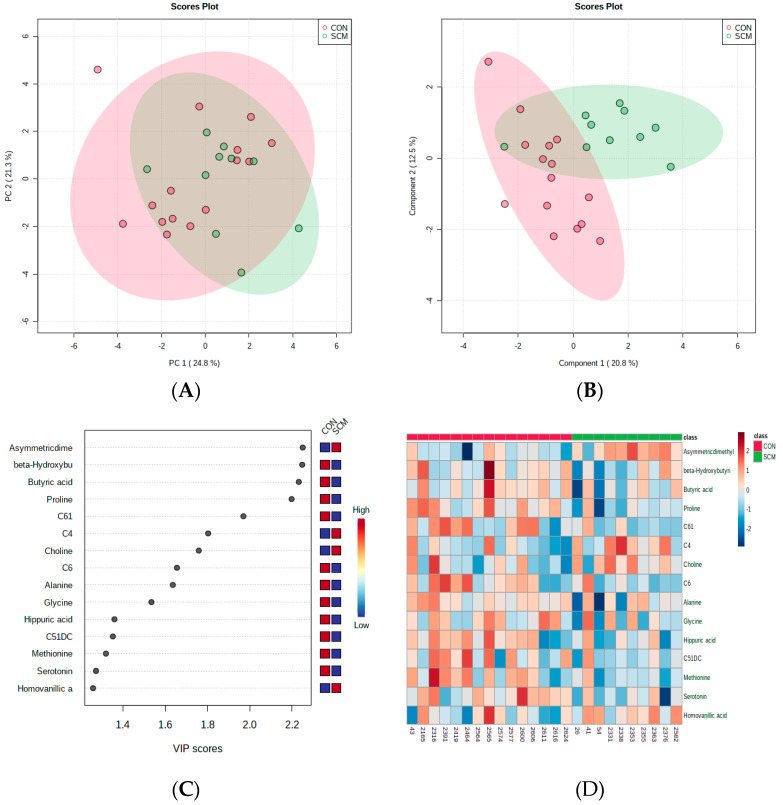
(**A**) Principal Component Analysis (PCA) differentiating CON from pre-SCM metabolite profiles 8 wks pre-parturition; (**B**) partial Least Squares-Discriminant Analysis (PLS-DA) classification of CON vs. pre-SCM metabolites (*p* > 0.05); (**C**) top 15 metabolites by Variable Importance in Projection (VIP) score; (**D**) heatmap based on PLS-DA for variable examination.

Multivariate statistical methods, including PCA and PLS-DA, were employed to cluster and differentiate the two cow groups. The PCA analysis did not exhibit clear separation between CON and pre-SCM cows at 8 wks before parturition; nevertheless, the PLS-DA graph distinctly distinguished the two groups (Figure 1A,B). VIP scores were utilized to assess variable significance in the PLS-DA. The VIP plot displayed the top 15 metabolites that distinguished cows with SCM from healthy ones (Figure 1C). The metabolite with the highest VIP score, 2.2, was asymmetric dimethylarginine (ADMA). Homovanillic acid (HVA) ranked second among the most important metabolites. Interestingly, this is the first association of HVA with pre-SCM. Both ADMA and HVA levels consistently increased in the urine of pre-SCM cows at 8 wks and 4 wks prepartum.

**Figure 2:**
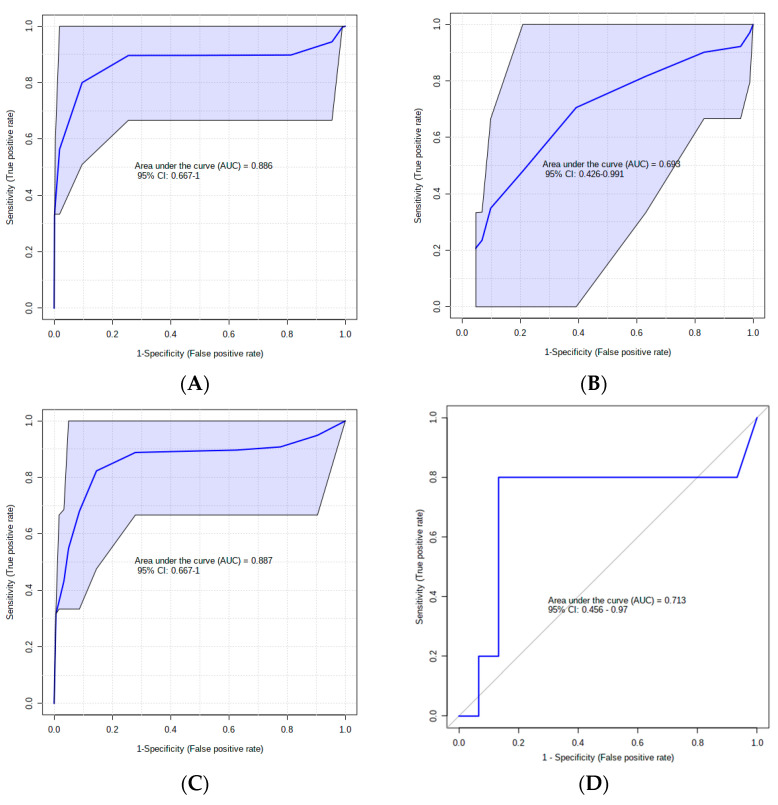
(**A**) Area Under the Curve (AUC) for five most significant metabolites from VIP; (**B**) AUC for optimal marker model via feature selection, focusing on 10 top metabolites; (**C**) Linear Support Vector Machine (SVM) biomarker panel (Asymmetric Dimethylarginine, Proline, Leucine, and Homovanillic Acid) performance (AUC = 0.88, *p* = 0.02); (**D**) Receiver Operating Characteristic (ROC) curve analysis with 10-fold cross-validation for four amino acids (AUC = 0.71, *p* = 0.04).

Figure 2 shows the predictive performance of urinary metabolites identified as potential SCM biomarkers. With an AUC = 0.88 and *p* = 0.02 for the top five metabolites from the VIP plot, these five metabolites demonstrated good predictive performance parameters (Figure 2A). Overall, the multivariate analysis produced the highest area under the ROC (AUROC) curve for the top ten most significant features (Figure 2B). To create a high-performing panel of predictive biomarkers, we chose specific metabolites that were easily validated. For each prepartum sampling period, a default linear support vector machine (SVM) model and a logistic regression algorithm were constructed. Both had a permutation test value of *p* ≤ 0.05 after 1000 randomized permutations (Figure 2C,D).

**Figure 3:**
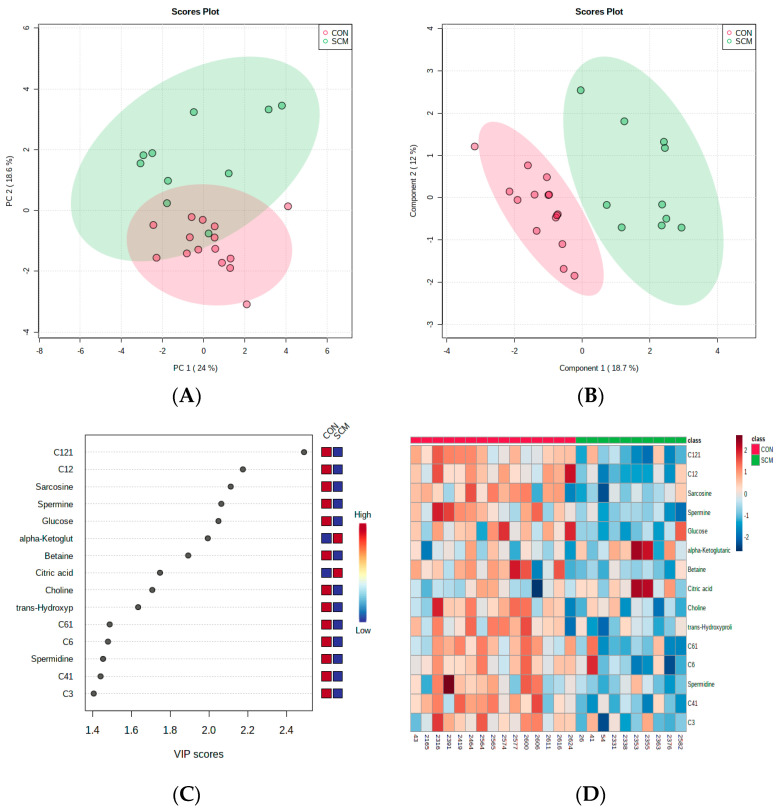
(**A**) Principal Component Analysis (PCA) differentiating control (CON) from pre-Subclinical Mastitis (pre-SCM) metabolite profiles 8 wks pre-parturition; (**B**) Partial Least Squares-Discriminant Analysis (PLS-DA) classification of CON vs. pre-SCM metabolites (*p* > 0.05); (**C**) top 15 metabolites by Variable Importance in Projection (VIP) score; (**D**) heatmap based on PLS-DA for variable examination.

**Figure 4:**
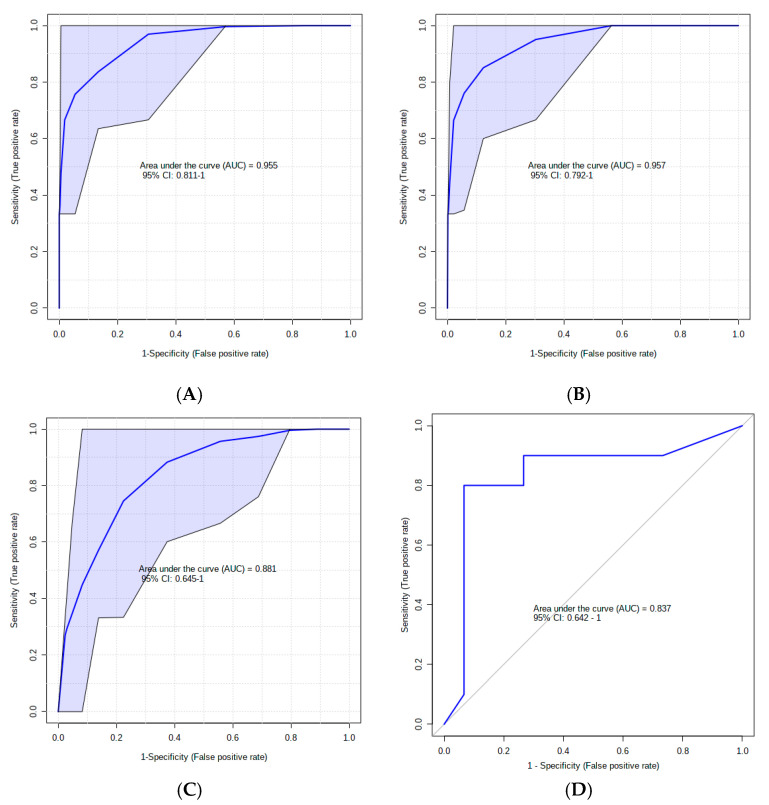
(**A**) Area Under the Curve (AUC) for the five most significant metabolites from Variable Importance in Projection (VIP); (**B**) AUC for the optimal biomarker model via feature selection, focusing on the top 10 metabolites; (**C**) Linear Support Vector Machine (SVM) model’s AUC for Asymmetric Dimethylarginine, Proline, Leucine, and Homovanillic Acid (AUC = 0.88, *p* = 0.03); (**D**) AUC for the logistic regression model analyzing these metabolites (AUC = 0.83, *p* = 0.05).

Our multivariate analysis, in contrast, demonstrated a more pronounced differentiation of pre-SCM and CON cows at 4 wks prior to calving (Figure 3A,B). Figure 3C,D highlights the metabolites that had the greatest impact in distinguishing these two groups. Six acylcarnitines (ACs) showed lower levels in pre-SCM cows at 4 wks, as indicated by the VIP plot, while two organic acids (OAs), alpha-ketoglutaric acid and citric acid, exhibited high VIP scores of >1.5 in pre-SCM. The top five metabolites from the VIP plot yielded an AUC of 0.95 (*p* = 0.009), and the model with the best classification, based on cross-validation, included all ten high-scoring metabolites (Figure 4A,B). We selected four metabolites for linear SVM and regression analysis due to their consistent performance throughout the model validation analysis (Figure 4C,D). The logistic regression model yielded a statistically significant result (*p* = 0.05), while the linear SVM model produced a highly significant result (*p* = 0.03). 

**Figure 5:**
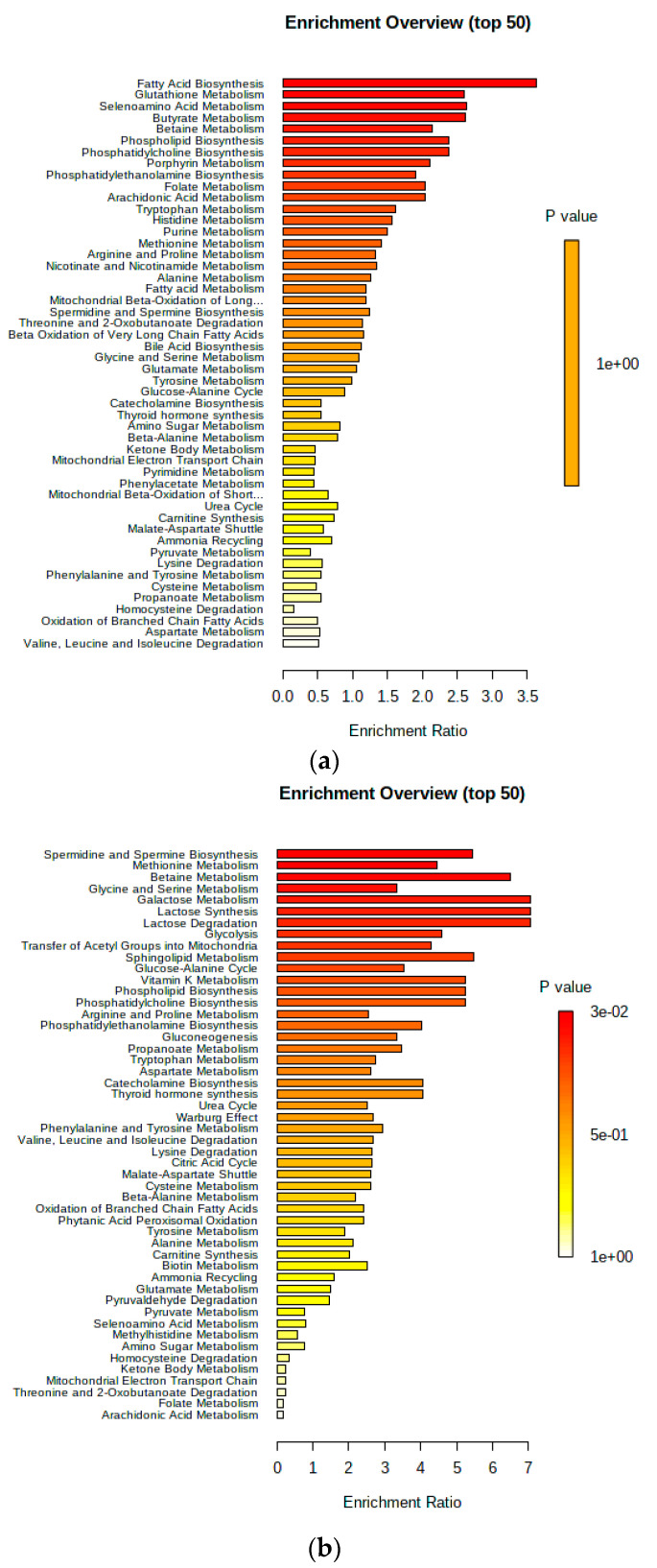
Overview diagrams showcasing the outcomes of quantitative enrichment analysis (QEA) performed via Metabolite Set Enrichment Analysis (MSEA) at two distinct intervals: (**a**) 8 wks and (**b**) 4 wks prepartum. The significance bar for *p* values illustrates the degree of enrichment significance across various metabolite collections at these intervals. At 8 wks (**a**), a prominent red section towards the top, marked by a *p* value of 0.03 (3 × 10^−2^), indicates a statistically significant enrichment. Conversely, at 4 wks (**b**), a yellow section towards the bottom, denoted by a *p* value of 1.0 (1 × 10^0^), indicates an absence of significant enrichment.

The findings from the quantitative enrichment analysis (QEA) conducted in MetaboAnalyst 4.0 are presented in Figure 5. At week 8 before parturition, no metabolic pathways exhibited significant alterations with a Holm value of *p* < 0.05 (Figure 5a). However, at 4 wks prior to calving, only one pathway associated with spermidine and spermine biosynthesis showed statistical significance (Holm *p* < 0.05) (Figure 5b). Additionally, urinary metabolites including spermine, methionine, spermidine, ornithine, and putrescine were significantly different (Holm *p* = 0.02) between the two groups of cows at 4 wks, while no significant metabolic pathways were identified at 8 wks before calving.

## 4. Discussion

In this study, our aim was to investigate whether prepartum urinary metabolites could serve as predictive markers for subclinical mastitis (SCM) in postpartum dairy cows, building on our previous research using serum metabolite biomarkers [9]. Specifically, we sought to determine if dairy cows prone to SCM exhibit distinct urinary metabotypes compared to healthy CON cows at 8 wks and 4 wks before calving, before the onset of postpartum SCM diagnosis. Our findings demonstrated that pre-SCM cows displayed unique urinary metabotypes that differentiated them from healthy (CON) cows as early as 8 wks and 4 wks prepartum. To differentiate between pre-SCM and healthy CON cows, somatic cell count (SCC) measurements in pre- and post-partum milk were utilized. A threshold of >200,000 SCC/mL of milk was applied for diagnosing SCM cows, while a SCC value of <200,000 was used for identifying healthy cows [1].

### 4.1. Urinary AC Alterations in Pre-SCM Cows

Among the metabolite species that differentiated pre-SCM cows from CON cows, urinary short-chain acylcarnitines (ACs) were particularly significant. Acylcarnitines play a crucial role in transporting fatty acids into the mitochondria for β-oxidation [13]. Similar findings were previously reported by our laboratory in the urine of pre-SCM and pre-ketotic cows [6,14]. Elevated AC levels have been recognized as biomarkers for immune system activation [15]. These compounds are by-products of incomplete mitochondrial fatty acid oxidation and must be eliminated from the body due to their toxicity. Other studies have observed increased blood AC levels in dairy cows infused with intramammary lipopolysaccharide (LPS) [16]. In the same study, the authors noted that LPS administration led to the suppression of apolipoprotein B genes, acetyl-CoA acyltransferase-2 (ACAA2), and hydroxymethylglutaryl-CoA synthase (HMGCS2) in hepatocytes, which are involved in β-oxidation, resulting in systemic accumulation of ACs. Indeed, our laboratory previously reported that pre-SCM cows exhibited a chronic low-grade inflammatory state during the dry-off period and the week preceding the postpartum disease diagnosis [4].

Interestingly, previous reports have indicated that individuals with high urinary acylcarnitines (ACs) may have methylmalonic acidemia (MMAemia) [17]. In our study, pre-SCM cows exhibited higher concentrations of methylmalonate in their urine and bloodstream, particularly −4 wks before parturition. In both humans and cattle, MMAemia is commonly caused by genetic errors or a deficiency in vitamin B12. Methylmalonate is produced as a by-product of branched-chain amino acid (BCAA) catabolism. It may be associated with defects in methylmalonyl-CoA mutase (MUT) or vitamin B12 synthesis, which hinder its entry into the Krebs cycle. Consequently, the body is unable to metabolize amino acids such as valine, isoleucine, threonine, methionine, and fatty acids, resulting in the accumulation of methylmalonic acid in the systemic circulation [18].

### 4.2. Changes in Urinary Amino Acids in Pre-SCM Cows

Urinary levels of branched-chain amino acids (BCAAs), including valine, leucine, and isoleucine, were found to be elevated in the pre-SCM group compared to healthy cows at both prepartum time points in our study. Similar findings were reported by our lab in a previous study involving pre-SCM and pre-lame cows [6,19]. Interestingly, BCAAs were also found to be higher in the serum of the same cows 8 wks before parturition.

These findings suggest that, for reasons yet unknown, the host fails to fully utilize BCAAs, resulting in their excretion in the urine. This phenomenon may resemble the pathogenesis of diabetic kidney disease (DKD) in humans. Previous research has demonstrated that BCAAs can increase the production of reactive oxygen species (ROS) in cultured peripheral blood mononuclear cells (PBMCs) by activating NADPH oxidase, mitochondria, and the Akt-mTOR signaling pathway [20]. BCAAs also stimulate the NF-kB pathway in these cells, leading to the release of proinflammatory cytokines such as interleukin 6 (IL-6) and tumor necrosis factor (TNF), as well as intracellular adhesion molecule-1 (ICAM-1) or CD40L, promoting PBMC migration. Based on these findings, it is hypothesized that elevated BCAA concentrations may contribute to proinflammatory responses and oxidative stress in various diseases. We suspect that the high concentrations of BCAAs in the blood and urine of our pre-SCM cows contribute to a systemic inflammatory state, as previously reported by our lab in pre-SCM cows [4].

One possible explanation for the excretion of BCAAs and several other amino acid (AA) species in urine, rather than their reabsorption into the systemic circulation, could be an increase in proteocatabolism in skeletal muscles to support the inflammatory response [21]. This could result in an excess of BCAAs that exceeds the resorptive capacity, or a deficiency of electrolytes that could bind with BCAAs. The release of proinflammatory cytokines like TNF or the translocation of LPS into the systemic circulation during chronic inflammatory states can lead to skeletal muscle protein loss due to increased muscle proteolysis and decreased muscle protein synthesis [22]. This hypothesis is supported by our finding that two urinary AAs, histidine and methyl-histidine, were elevated in pre-SCM cows 4 wks before parturition. These AAs have been associated with increased proteolysis of muscle protein [23]. In humans, increased histidine proteolysis and urinary excretion are linked to higher concentrations of IL-6, C-reactive protein (CRP), and elevated levels of ROS [23]. Similar findings have been reported in rodents [24]. Histidine exhibits anti-inflammatory effects in response to LPS challenge, including ROS scavenging and inhibition of IL-8 and NF-kB secretion [25]. Conversely, high urinary excretion of histidine and methyl-histidine may indicate a host response to a potential subclinical bacterial infection of the udder during the dry-off period.

Another notable finding was the elevated urinary concentrations of arginine (at 8 wks prepartum) and two closely related functional components, ADMA and TDMA (total dimethylarginine), in the urine of pre-SCM cows. These findings align with our previous observations in pre-SCM and pre-lameness cows [6], where we detected increased arginine levels in the serum of pre-SCM cows at 4 wks prepartum. Arginine, an essential amino acid, plays a crucial role in nitric oxide (NO) production, polyamine synthesis, proline formation, and immune system stimulation [26]. Arginine catabolism has been shown to increase during inflammatory conditions.

Interestingly, arginine has been found to attenuate LPS-induced inflammation in mammary epithelial cells by reducing the release of IL-1β, IL-6, and TNF, and enhancing mTOR signaling [27]. Furthermore, arginine infusion in cows during early lactation has been associated with lower levels of TNF and Hp, increased IgM concentrations, enhanced total protein content, and improved antioxidant capacity [28]. In a sheep study, LPS-induced inflammation was linked to increased arginine depletion in the liver and spleen [29]. The increased urinary excretion of arginine in pre-SCM cows may contribute to a proinflammatory state aimed at combating potential subclinical mastitis.

Notably, ADMA has gained significant attention due to its ability to inhibit nitric oxide synthase (NOS) activity [30]. Nitric oxide synthase is the enzyme responsible for converting arginine into nitric oxide (NO) and L-citrulline. ADMA is produced through the asymmetric demethylation of arginine residues in specific proteins and is released during proteolysis. Elevated levels of free ADMA in the systemic circulation have been identified as a risk factor for morbidity and mortality in humans [31]. This risk is associated with the inhibition of NO secretion, which plays a crucial role in host defense and serves as a major killing mechanism for macrophages. The excretion of ADMA in urine may represent a host response to eliminate this compound, which inhibits immune cell killing activity.

Regarding our findings, the concentrations of choline and betaine in the urine of pre-SCM cows were higher at 8 wks postpartum. Only urinary choline exhibited higher levels in these cows at 4 wks prior to parturition. Choline, an essential nutrient and amine, participates in acetylcholine synthesis and serves as a methyl group donor in the liver and kidneys [32]. Choline is also involved in phosphatidylcholine production [33]. Researchers investigating choline in murine macrophages observed increased choline uptake during the polarization of primary bone marrow macrophages with LPS [34].

Choline uptake has also been associated with IL-1-dependent inflammation mediated by macrophages. Both choline and betaine have demonstrated anti-inflammatory properties. For instance, a study revealed that injecting mice with 50 mg/kg of choline intraperitoneally prior to LPS administration resulted in reduced systemic levels of TNF [35]. Additionally, the same authors demonstrated that choline inhibited TNF release in human macrophages.

Betaine, which plays a role in one-carbon metabolism and acts as a methyl group donor, has been shown to suppress the NF-κB pathway and its associated genes, including TNF, vascular cell adhesion molecule-1, intracellular cell adhesion molecule-1, inducible nitric oxide synthase, and cyclooxygenase-2 [36].

It may seem peculiar that the host excretes anti-inflammatory compounds in the urine. However, it should be noted that both choline and betaine exhibit higher concentrations in the serum of the same cows at −8 wks prepartum (unpublished results). These findings support our previously proposed hypothesis that the host’s metabolic response, as reflected in the urinary excretion of choline and betaine, supports a proinflammatory response against the presence of potential subclinical infection in the udder.

### 4.3. Changes in the Urinary Carbohydrate and Organic Acid Species in Pre-SCM Cows

The concentration of glucose in the urine of pre-SCM cows exhibited an elevation at the onset of the dry-off period (8 wks prepartum) compared to 4 wks prepartum. Serum glucose concentrations tended to be higher in the pre-SCM cows at 8 wks prepartum (unpublished results). Despite playing a crucial role as an energy substrate for the host, elevated glucose levels have been associated with impaired immune functions and increased susceptibility to bacterial infections. For instance, impaired natural killer (NK) cell functions were reported in type 2 diabetes patients compared to healthy controls and prediabetic patients [37]. Recent studies also demonstrated impaired neutrophil migration to the site of inflammation in the lungs of diabetic (hyperglycemic) rats [38]. Furthermore, chronic hyperglycemia has been shown to increase neutrophil basal ROS production and susceptibility to infection due to decreased neutrophil responses [39]. Considering the potential detrimental effects of high blood glucose concentrations on immune responses, particularly neutrophil and NK cell functions, it appears that the host increases urinary excretion to prevent glucose-related impairment of immune responses.

To the best of our knowledge, this study is the first to report elevated concentrations of urinary homovanillic acid (HVA) in pre-SCM cows. Homovanillic acid, a downstream metabolite of tyrosine, exhibited increased levels at both sampling times, with stronger predictive abilities at −8 wks prepartum. Elevated HVA is a well-established urine biomarker for various human tumors, metabolic disorders, and neurological disorders [40]. It is a catabolite of catecholamines, particularly dopamine. Catecholamines are considered coping hormones in stressful situations and significant activators of lipolysis and glycogenolysis for energy support [41]. Homovanillic acid has been found to activate the immune system in humans, and lower concentrations correlate with host survival [42]. The reason for the increased HVA in the urine of pre-SCM cows remains unclear, suggesting the diverse range of mastitis pathomechanisms.

At 4 wks prepartum, there was a substantial disparity in urinary citrate concentration between pre-SCM cows (21-fold higher) and CON cows. Citrate is an important metabolite in the Krebs cycle, produced in mitochondria and then transferred to the cytosol of macrophages, where it is considered essential for the pro-inflammatory response [43]. Under normal conditions, cytosolic citrate is converted into acetyl-CoA and oxaloacetate. Acetyl-CoA is involved in fatty acid synthesis, while oxaloacetate contributes to the production of reactive oxygen species (ROS), crucial in combating pathogenic bacteria. Research is increasingly highlighting the significance of citrate in regulating immune cell response. Citrate has been linked to macrophage production of several key proinflammatory mediators, including nitric oxide (NO), ROS, and prostaglandin E2 (PGE2) [44]. In fact, inhibiting the citrate carrier protein (CIC) significantly decreases the production of NO, ROS, and PGE2 [45]. Furthermore, LPS treatment has been shown to increase mitochondrial CIC in LPS-induced macrophages [45]. The reasons why pre-SCM cows excrete exceptionally high amounts of citrate in the urine remain important questions for this study. The answer is yet to be determined. However, this finding supports our hypothesis that pre-SCM cows are attempting to mount an inflammatory response, and the significant urinary excretion of citrate, known for its inhibitory effects on proinflammatory mediators, suggests an effort to maintain the inflammatory response within specific limits.

Notably, there were significant differences in urinary polyamine concentrations between the cow groups. Pre-SCM cows exhibited higher levels of spermidine and putrescine at 8 wks prepartum compared to CON cows. Additionally, pre-SCM cows had elevated levels of urinary spermine at 4 wks prepartum. Polyamines, including spermidine, putrescine, and spermine, derived from ornithine, play vital physiological roles in mammals [46]. They contribute to DNA and protein synthesis, cell proliferation, and differentiation. Moreover, polyamines act as ROS scavengers, safeguarding DNA, proteins, and lipids from oxidative damage. These compounds are known for their anti-inflammatory and antioxidant properties [47]. Some studies have investigated the effects of polyamines on acute, subacute, and chronic inflammation, revealing significant anti-inflammatory activity in these models [48]. Our data supports the notion that pre-SCM cows excrete higher amounts of polyamines, which possess anti-inflammatory properties, in their urine compared to CON cows. Based on our findings, it appears that pre-SCM cows release various anti-inflammatory metabolites in their urine. This aligns with our hypothesis that pre-SCM cows are attempting to mount an inflammatory response while simultaneously excreting multiple metabolites that may regulate and control the inflammatory process in the systemic circulation.

## 5. Conclusions

In summary, pre-SCM cows displayed altered concentrations of urinary metabolites associated with acylcarnitines, amino acids, carbohydrate, and organic acid metabolism. The increased excretion of acylcarnitines and clearance of several amino acids, such as valine, leucine, isoleucine, histidine, methyl-histidine, arginine, as well as choline and betaine, might contribute to a systemic inflammatory response triggered by a subclinical udder infection. Other metabolites, including ADMA, TDMA, glucose, and citrate, were excreted in the urine, potentially exerting inhibitory effects on immune cells and limiting the cow’s response to the disease agent(s). Our findings suggest that various metabolites are excreted in the urine to mitigate the inflammatory response, while others are retained to support it. Multivariate analysis demonstrated distinct separation between the cow groups at 4 wks prepartum, but not at 8 wks prepartum. Two useful panels of urinary metabolites were constructed to predict SCM, exhibiting high prediction accuracy. At 8 wks prepartum, ADMA, MMA, spermidine, and citrate, while at 4 wks prepartum, ADMA, leucine, proline, and HVA were identified as potential metabolites for predicting the risk of SCM starting one week prior to the dry-off period.

## Figures and Tables

**Table 1:** Ingredients of the prepartum diet for the dry-off cows [9].

Ingredient	Weight/Cow (kg)	DM (%)	Final DMI (kg) ^1^
Hay	5.50	85.14%	4.68
Oats	5.75	36.20%	2.08
Corn	8.84	30.30%	2.68
Protein	2.00	93.00%	1.86
Ground Barley	0.75	97.26%	0.66
Minerals	0.42	97.26%	0.41
Total	23.36	53.17%	12.37

^1^ Dry Matter Intake (DMI) is calculated based on the DM% over the offered amount (kg) of feed. Daily DMI is formulated to be 2% of a cow’s body weight.

**Table 2:** Feed ingredients on a dry-matter basis for cows during early lactation [9].

Ingredient	Weight/Cow (kg)	DM (%)	Final DMI (kg)
Hay dairy	2.50	88.50	2.21
Grass silage	10.75	31.80	3.42
Oats	5.99	36.20	2.17
Barley-Dakota	11.50	40.00	4.80
Corn	13.52	31.50	4.26
Whey	2.75	17.00	0.47
Protein	4.75	93.30	4.43
Energy dairy	4.25	88.00	3.74
Ground Barley	1.75	88.00	1.54
Mineral & Fat	1.26	97.26	1.23
Total	59.02	47.56	28.07

**Table 3:** Concentration of urinary metabolites (MEAN ± SEM) in pre-subclinical mastitis cows (pre-SCM, n = 10) and healthy controls (CON, n = 15) at −8 wks prior to parturition as identified by LC-MS/MS.

Metabolites (μM)	MEAN ± SEM		Fold Change	SCM/CON	*p*-Value
	Pre-SCM ^1^ (n = 10)	CON ^2^ (n = 15)			
Creatinine	13,903 ± 1679	8732 ± 1477	1.59	up	0.01
Glycine	302 ± 169	196 ± 149	1.54	up	0.78
Alanine	265 ± 60	171 ± 52.8	1.55	up	0.48
Serine	103 ± 17.3	56 ± 15.2	1.84	up	0.11
Histamine	0.1315 ± 0.0244	0.0915 ± 0.0214	1.44	up	0.28
Proline	6.14 ± 1.56	5.54 ± 1.37	1.11	up	0.42
Valine	18.48 ± 3.41	8.04 ± 3	2.30	up	0.02
Threonine	84.4 ± 18.3	46.4 ± 16.1	1.82	up	0.2
Taurine	655 ± 211	420 ± 186	1.56	up	0.55
Putrescine	0.724 ± 0.144	0.299 ± 0.127	2.42	up	0.01
trans-Hydroxyproline	1.69 ± 0.598	1 ± 0.526	1.69	up	0.86
Leucine	12.4 ± 1.94	6.3 ± 1.7	1.97	up	0.01
Isoleucine	19.34 ± 6.6	7.83 ± 5.8	2.47	up	0.28
Asparagine	13.1 ± 1.79	8.47 ± 1.57	1.55	up	0.05
Aspartic acid	174 ± 36.8	127 ± 32.4	1.37	up	0.43
Glutamine	347 ± 75.1	154 ± 66	2.25	up	0.15
Glutamic acid	107 ± 31.2	80 ± 27.5	1.34	up	0.64
Methionine	3.33 ± 0.29	2.7 ± 0.255	1.23	up	0.06
Histidine	108.2 ± 24.6	53.1 ± 21.6	2.04	up	0.17
alpha-Aminoadipic acid	135 ± 26.6	72 ± 23.4	1.88	up	0.25
Phenylalanine	16.38 ± 2.72	9.87 ± 2.4	1.66	up	0.09
Methionine-sulfoxide	3.81 ± 0.948	1.86 ± 0.834	2.05	up	0.17
Arginine	15.46 ± 2.05	8.15 ± 1.8	1.90	up	0.01
Acetyl-ornithine	76.1 ± 12.5	44 ± 11	1.73	up	0.08
Citrulline	8.54 ± 2.36	3.5 ± 2.07	2.44	up	0.07
Serotonin	1.79 ± 0.297	1.37 ± 0.262	1.31	up	0.44
Tyrosine	27.4 ± 5	16.4 ± 4.4	1.67	up	0.11
Asymmetric dimethylarginine	9.48 ± 1.21	2.61 ± 1.07	3.63	up	<0.001
Total dimethylarginine	33.8 ± 4.03	18.3 ± 3.55	1.85	up	0.007
Tryptophan	36 ± 8.65	19.3 ± 7.61	1.87	up	0.28
Kynurenine	1.585 ± 0.449	0.967 ± 0.395	1.64	up	0.71
Carnosine	21.7 ± 4.39	15.5 ± 3.86	1.40	up	0.4
Ornithine	25.8 ± 4.44	14 ± 3.91	1.84	up	0.07
Lysine	82.1 ± 14.1	43.4 ± 12.4	1.89	up	0.08
Spermidine	0.2248 ± 0.0871	0.0746 ± 0.0766	3.01	up	0.05
Spermine	0.0868 ± 0.0171	0.0791 ± 0.015	1.10	up	0.43
Sarcosine	4.69 ± 1.17	1.27 ± 1.03	3.69	up	0.17
Tyramine	0.183 ± 0.042	0.111 ± 0.0501	1.65	up	0.26
Creatine	5098 ± 663	2354 ± 583	2.17	up	0.001
Betaine	265.2 ± 67.6	74.9 ± 59.4	3.54	up	0.02
Choline	92.8 ± 21.5	32.1 ± 18.9	2.89	up	0.007
Trimethylamine N-oxide	6150 ± 1895	3677 ± 1667	1.67	up	0.38
Methylhistidine	198 ± 52.3	370 ± 59.4	0.54	down	0.05
Lactic acid	125 ± 53.9	112 ± 47.4	1.12	up	0.93
beta-Hydroxybutyric acid	400 ± 471	416 ± 415	0.96	down	0.47
alpha-Ketoglutaric acid	25.7 ± 45.1	34.8 ± 39.7	0.74	down	0.47
Citric acid	856 ± 801	778 ± 684	1.10	up	0.84
Butyric acid	28.4 ± 14.7	29.5 ± 12.9	0.96	down	0.35
p-hydroxyhippuric acid	36.7 ± 14.1	37.9 ± 12.4	0.97	down	0.93
Succinic acid	30.3 ± 9.86	20.6 ± 8.67	1.47	up	0.69
Pyruvic acid	8.71 ± 1.79	6.33 ± 1.58	1.38	up	0.16
Isobutyric acid	7.23 ± 1.66	5.56 ± 1.46	1.30	up	0.59
Hippuric acid	14,438 ± 2073	13,225 ± 1823	1.09	up	0.65
Methylmalonic acid	29.4 ± 6.68	17.9 ± 5.87	1.64	up	0.31
Homovanillic acid	14.67 ± 1.46	8.83 ± 1.28	1.66	up	<0.001
Indole acetic acid	67.5 ± 20.7	51.3 ± 18.2	1.32	up	0.96
Uric acid	5014 ± 883	4279 ± 776	1.17	up	0.44
Glucose	3369 ± 462	1955 ± 406	1.72	up	0.002
C0	2.516 ± 0.386	0.893 ± 0.339	2.82	up	0.01
C2	0.714 ± 0.0915	0.305 ± 0.0805	2.34	up	0.001
C3:1	0.0319 ± 0.00428	0.0258 ± 0.00376	1.24	up	0.02
C3	0.0402 ± 0.00722	0.037 ± 0.00635	1.09	up	0.33
C4:1	0.0871 ± 0.00819	0.0631 ± 0.00721	1.38	up	0.04
C4	0.58 ± 0.1096	0.129 ± 0.0964	4.50	up	0.002
C3OH	0.0855 ± 0.0092	0.0642 ± 0.00809	1.33	up	0.11
C5:1	0.251 ± 0.0226	0.147 ± 0.0199	1.71	up	0.001
C5	0.1598 ± 0.0273	0.0929 ± 0.024	1.72	up	0.18
C4OH	0.0898 ± 0.00984	0.0653 ± 0.00865	1.38	up	0.05
C6:1	0.057 ± 0.0122	0.0817 ± 0.0108	0.70	down	0.27
C6	0.072 ± 0.0134	0.0872 ± 0.0118	0.83	down	0.77
C5OH	0.1372 ± 0.0131	0.0849 ± 0.0115	1.62	up	0.002
C5:1DC	0.0438 ± 0.00413	0.0377 ± 0.00363	1.16	up	0.23
C5DC	0.0469 ± 0.00595	0.0323 ± 0.00523	1.45	up	0.03
C8	0.0556 ± 0.00556	0.0356 ± 0.00489	1.56	up	0.003
C5MDC	0.0514 ± 0.00366	0.0466 ± 0.00322	1.10	up	0.02
C9	0.147 ± 0.023	0.106 ± 0.0203	1.39	up	0.21
C7DC	0.0437 ± 0.00899	0.0385 ± 0.00791	1.14	up	0.5
C10:2	0.0578 ± 0.00732	0.0437 ± 0.00644	1.32	up	0.19
C10:1	0.171 ± 0.0177	0.15 ± 0.0156	1.14	up	0.15
C10	0.135 ± 0.0125	0.104 ± 0.011	1.30	up	0.01
C12:1	0.14 ± 0.0387	0.127 ± 0.034	1.10	up	0.43
C12	0.1037 ± 0.00933	0.0943 ± 0.0082	1.10	up	0.01

^1^ pre-SCM = SCM = cows that were sampled before being classified as SCM. ^2^ CON = healthy cows.

**Table 4:** Metabolite concentration of urine metabolites MEAN ± SEM in pre-subclinical mastitis (pre-SCM, n = 10) and healthy controls (CON, n = 15) at −4 wks prior to parturition as identified by LC-MS/MS.

Metabolites (μM)	MEAN ± SEM		Fold Change	SCM/CON	*p*-Value
	Pre-SCM ^1^ (n = 10)	CON ^2^ (n = 15)			
Creatinine	14,300 ± 1521	10,569 ± 1140	1.35	up	0.01
Glycine	105 ± 28.3	67.5 ± 21.2	1.56	up	0.1
Alanine	97.5 ± 11.7	82.5 ± 8.8	1.18	up	0.23
Serine	77 ± 10.39	69 ± 7.79	1.12	up	0.1
Histamine	0.0994 ± 0.0154	0.0642 ± 0.0116	1.55	up	0.12
Proline	3.63 ± 0.531	3.94 ± 0.398	0.92	down	0.44
Valine	11.1 ± 1.185	10.3 ± 0.888	1.08	up	0.28
Threonine	70.7 ± 10.08	50.9 ± 7.56	1.39	up	0.01
Taurine	439 ± 136	395 ± 102	1.11	up	0.71
Putrescine	0.915 ± 0.503	1.167 ± 0.377	0.78	down	0.84
trans-Hydroxyproline	1.22 ± 0.425	1.92 ± 0.318	0.64	down	0.3
Leucine	9.1 ± 0.99	9.13 ± 0.742	1.00	up	0.38
Isoleucine	7.72 ± 0.745	5.98 ± 0.558	1.29	up	0.007
Asparagine	13.46 ± 1.48	9.67 ± 1.11	1.39	up	0.01
Aspartic acid	190 ± 31.7	131 ± 23.7	1.45	up	0.17
Glutamine	285 ± 46.8	206 ± 35.1	1.38	up	0.04
Glutamic acid	78.6 ± 11.7	53.6 ± 8.8	1.47	up	0.09
Methionine	3.34 ± 0.252	3.25 ± 0.189	1.03	up	0.4
Histidine	76.6 ± 9.85	60.4 ± 7.38	1.27	up	0.05
alpha-Aminoadipic acid	79.9 ± 14.5	72.9 ± 10.8	1.10	up	0.31
Phenylalanine	13.1 ± 1.08	10.4 ± 0.81	1.26	up	0.03
Methionine-sulfoxide	3.12 ± 0.478	3.13 ± 0.358	1.00	up	0.83
Arginine	11.3 ± 1.316	10.1 ± 0.986	1.12	up	0.48
Acetyl-ornithine	57 ± 6.82	47.4 ± 5.11	1.20	up	0.08
Citrulline	3.47 ± 1.265	6.94 ± 0.948	0.50	down	0.17
Serotonin	1.66 ± 0.202	1.33 ± 0.151	1.25	up	0.13
Tyrosine	19.4 ± 2.38	20 ± 1.78	0.97	down	0.8
Asymmetric dimethylarginine	8.39 ± 1.024	6.26 ± 0.768	1.34	up	0.02
Total dimethylarginine	36.2 ± 3.36	26.4 ± 2.52	1.37	up	0.008
Tryptophan	19.8 ± 3.17	17.8 ± 2.38	1.11	up	0.58
Kynurenine	0.735 ± 0.0835	0.696 ± 0.0625	1.06	up	0.94
Carnosine	14.7 ± 1.61	11.1 ± 1.21	1.32	up	0.03
Ornithine	16.7 ± 2.1	15.5 ± 1.58	1.08	up	0.6
Lysine	59.7 ± 5.68	48.5 ± 4.26	1.23	up	0.07
Spermidine	0.0772 ± 0.0256	0.1113 ± 0.0192	0.69	down	0.36
Spermine	0.1219 ± 0.0113	0.0596 ± 0.015	2.05	up	0.008
Sarcosine	3.04 ± 2.02	6.92 ± 1.51	0.44	down	0.04
Tyramine	0.133 ± 0.0237	0.113 ± 0.0188	1.18	up	0.82
Creatine	5737 ± 1558	6460 ± 1168	0.89	down	0.69
Betaine	134 ± 95.7	364 ± 71.7	0.37	down	0.06
Choline	18 ± 12.98	56.9 ± 9.73	0.32	down	0.05
Trimethylamine N-oxide	5083 ± 1353	1338 ± 1014	3.80	up	0.03
Methylhistidine	373 ± 33.7	246 ± 25.2	1.52	up	0.001
Lactic acid	256 ± 83.6	107 ± 62.6	2.39	up	0.13
beta-Hydroxybutyric acid	116 ± 60.2	135 ± 45.1	0.86	down	0.6
alpha-Ketoglutaric acid	129 ± 66.6	17.8 ± 49.9	7.27	up	0.07
Citric acid	1911 ± 723	91 ± 542	21.00	up	0.04
Butyric acid	11.59 ± 2.18	7.43 ± 1.63	1.56	up	0.09
p-hydroxyhippuric acid	44.9 ± 17.2	53.9 ± 12.9	0.83	down	0.96
Succinic acid	42.3 ± 14.7	17.9 ± 11	2.36	up	0.22
Pyruvic acid	21.87 ± 6.85	7.63 ± 5.13	2.87	up	0.08
Isobutyric acid	5.14 ± 0.753	2.71 ± 0.564	1.90	up	0.009
Hippuric acid	20,896 ± 3944	17,503 ± 2955	1.19	up	0.32
Methylmalonic acid	25.2 ± 4.18	10.5 ± 3.13	2.40	up	0.001
Homovanillic acid	13.55 ± 1.88	8.01 ± 1.41	1.69	up	0.03
Indole acetic acid	58.5 ± 9.75	36.4 ± 7.3	1.61	up	0.06
Uric acid	4707 ± 748	3036 ± 561	1.55	up	0.05
Glucose	387 ± 555	973 ± 416	0.40	down	0.35
C0	1.77 ± 0.167	1.15 ± 0.125	1.54	up	0.01
C2	0.685 ± 0.1239	0.685 ± 0.0928	1.00	up	0.75
C3:1	0.0501 ± 0.00367	0.047 ± 0.00275	1.07	up	0.9
C3	0.0492 ± 0.00432	0.0573 ± 0.00324	0.86	down	0.17
C4:1	0.0729 ± 0.0089	0.0767 ± 0.00667	0.95	down	0.86
C4	0.484 ± 0.0904	0.423 ± 0.0677	1.14	up	0.24
C3OH	0.0695 ± 0.00729	0.0714 ± 0.00546	0.97	down	0.83
C5:1	0.259 ± 0.0338	0.152 ± 0.0253	1.70	up	0.005
C5	0.155 ± 0.025	0.154 ± 0.0187	1.01	up	0.88
C4OH	0.0785 ± 0.00747	0.0739 ± 0.0056	1.06	down	0.52
C6:1	0.0691 ± 0.0081	0.0841 ± 0.00607	0.82	down	0.28
C6	0.0857 ± 0.0145	0.1026 ± 0.0108	0.84	down	0.61
C5OH	0.14 ± 0.0152	0.109 ± 0.0114	1.28	up	0.02
C5:1DC	0.0453 ± 0.00418	0.0349 ± 0.00313	1.30	up	0.07
C5DC	0.048 ± 0.00412	0.0282 ± 0.00309	1.70	up	<0.001
C8	0.0483 ± 0.00476	0.0428 ± 0.00357	1.13	up	0.33
C5MDC	0.0483 ± 0.0046	0.0482 ± 0.00345	1.00	up	0.79
C9	0.152 ± 0.0188	0.11 ± 0.0141	1.38	up	0.04
C7DC	0.0488 ± 0.00611	0.0281 ± 0.00458	1.74	up	0.005
C10:2	0.0466 ± 0.00574	0.0514 ± 0.0043	0.91	down	0.96
C10:1	0.204 ± 0.0213	0.176 ± 0.016	1.16	up	0.23
C10	0.12 ± 0.0151	0.136 ± 0.0113	0.88	down	0.84
C12:1	0.083 ± 0.0315	0.229 ± 0.0236	0.36	down	0.002
C12	0.0444 ± 0.0184	0.091 ± 0.0138	0.49	up	0.04

^1^ pre-SCM = SCM = cows that were sampled before being classified as SCM. ^2^ CON = healthy cows.

## Data Availability

The data presented in this study are confidential and not available to the public for intellectual property development reasons.
